# Struggling to Perform the State: The Politics of Bread in the Syrian Civil War

**DOI:** 10.1093/ips/olw026

**Published:** 2017-01-09

**Authors:** MartÍnez Ciro JosÉ, Brent Eng

**Affiliations:** 1 Department of Politics and International Studies, University of Cambridge; 2 Department of Anthropology, University of California, Berkeley

## Abstract

Recent studies of civil war have problematized frameworks that rely on a strict binary between state-sanctioned order and anarchy. This paper extends these insights and combines them with theories of performativity to examine welfare practices during the Syrian conflict (2011-2015). Specifically, we argue that conceptualizing the state as a construct—as an effect of power—can expand the study of civil war beyond its quantifiable aspects and embrace the performative dimensions of political life. By means of everyday, iterative acts such as welfare provision, competing groups summon the state, and the political order it seeks to enshrine, into existence: they make it both tangible and thinkable. During civil war, the ability to perform these prosaic acts becomes a matter of pressing military and political concern. Through close scrutiny of various cases, we dissect the impact of subsidized bread provision by the Assad regime, the Free Syrian Army, and armed Islamist groups as they struggle to perform the state. our aim is to bring attention to under-studied governance practices so as to analyze the otherwise opaque relations between welfare provision, military success, and civilian agency during Syria’s civil war.

Narratives of Syria’s war emphasize its martial elements. Commentators discuss the conflict mainly through the prisms of military strategy, weapons supply, territorial control, and external alliances. Most accounts choose to privilege bellicose affairs over the humdrum concerns of daily life, which are deemed humanitarian issues separate from the violent battles and geopolitical struggles said to comprise the “actual” politics of war. This portrayal of conflict is illusory: it disregards the majority of interactions that shape both life and politics in contemporary war zones, where “most people most of the time are interacting in non-violent ways” (Tilly 2003, 12). One result of prevalent depictions of civil war is that civilians are frequently rendered powerless. if they do appear, it is as pawns in a conflict fought by armed groups autonomous from the societies they struggle to control. In contrast, our analysis sheds light on how civilians—through their expectations, demands, and actions—shape governance during civil war. To do so, we explore one subsection of the welfare infrastructures operated by the Syrian government and various rebel groups: the provision of subsidized bread. Close scrutiny of this welfare service, which we conceptualize as crucial to “performing the state,” offers a valuable window into military and political developments during the Syrian civil war. It can also help reorient the study of conflict toward the lives of those whom it most intimately affects.

Although force and violence may be essential for any group to gain or maintain control over a territory or population, by themselves they are far from sustainable methods to winning anything but the shortest of conflicts. Evidence from other cases and close scrutiny of the Syrian conflict highlight the importance for belligerents of gaining collaboration, or at least acquiescence, from local populations (Mampilly 2011; Hassan and Weiss 2015). This is because discontented civilians pose a number of challenges to the calculations of fighting forces. Public opposition to governing entities can be conveyed peacefully by way of public protest or noncompliance with directives in the realm of taxation, agriculture, or conscription. It can also be expressed violently through local militias and collaboration with opponents. As competing military groups struggle to achieve support for their cause, the provision of services has proven critical to building consent and legitimacy among local populations (Kalyvas 2006, 124). Rebel groups in Sri Lanka, Colombia, and Sudan, among others, have constructed elaborate governance systems aimed at fulfilling the needs of civilians. Throughout Lebanon’s civil war (1975-89), various militias stepped in to provide a number of social services to populations under their control. These distributive efforts became intimately linked to the militias’ ability to maintain control over people and territory for extended periods of time (Khalaf 2002; Cammett and Issar 2010). By directly providing security, electricity, health services, and food—or indirectly doing so through access to benefits provided by humanitarian organizations (Matanock 2014; Schäferhoff 2014)—rebel groups can engender support and consolidate their control among the populations over whom they claim sovereignty (Kasfir 2005; Wood 2008; Mampilly 2011, 240). Government authorities and occupation forces have also recognized the importance of distributing public goods and services during wartime. Attempts to outperform their rivals through the superior provision of public services have long been a key element in counter-insurgency strategies (Kilcullen 2009; Staniland 2012). In brief, welfare provision plays crucial political functions amid the resource scarcities and physical insecurities that tend to characterize civil war.

Why do certain groups invest so much time and effort in providing welfare services to civilians under their control while others do not? How do residents exert influence over these processes, and what bearing do such provisions have on military and political developments? We explore these questions through a contextual approach to political analysis that scrutinizes Syria’s ongoing conflict (Tilly and Goodin 2006, 3-32). Following Mampilly (2011), we analyze governance during wartime as an evolutionary, iterative process whose outcomes cannot be predicted by a single variable or predominant factor. The provision of welfare can be understood only in relation to a variety of concurrent elements, demands, and resources that impact the behavior and decisions of rebel organizations, government authorities, and civilians.^1^ Crucial among these is the desire and ability of competing groups to “perform” like a state, as well as the reception of such practices among the populations they seek to rule.

This article begins by discussing predominant approaches to the study of governance during civil war before outlining why the provision of basic goods acts as a pivotal element of “state performance.” We then offer a brief history of food- related welfare programs in Syria, focusing on subsidized bread because of its socio-political importance before the war and throughout the current conflict (Martínez and Eng 2016). These first two sections will illuminate the micro processes through which certain welfare programs have become embedded in popular expectations and governmental practices so as to contextualize the manifestation of specific provisionary practices since late 2011. The article then examines wartime bread provision by the Assad regime and various opposition forces before dissecting their multivalent impact on civilian life and military developments. While we do not pretend to offer a comprehensive account of governance in regime and opposition-held areas, we highlight bread because of its political salience. Specifically, this article argues that the provision of welfare to civilians during Syria’s civil war has been as crucial to the long-term viability of any military force as its use of violence or access to material resources. Indeed, our research suggests that these dynamics are closely intertwined. We conclude by summarizing the impact of subsidized bread provision on the conflict before elaborating on the empirical and theoretical insights of our analysis.

The full scope of welfare provision in Syria is difficult to assess: the regime and its opponents regularly disseminate information, but the accuracy of such material is hard to confirm. Drawing on scholarly and personal knowledge of Syria, we attempt to assess wartime welfare practices without direct access to the country since 2011, due to security restrictions. From 2013 to 2015, we conducted 72 interviews by email, Skype, and telephone with civilians in 10 of the country’s 14 gover- norates. We additionally benefit from a robust qualitative sample of respondents collected outside Syria, which includes 136 interviews with Syrian refugees recently arrived in Jordan or Lebanon, many of whom remain in contact with relatives in the country and some of whom travel back to Syria regularly. We also draw on open-source material ranging from NGO and international organizations’ reports to newspapers and YouTube videos, as well as information and testimonies provided by activist networks operating in the country. To bolster our claims, we supplement our findings with those of various news sources. Almost all our informants inside Syria asked to remain anonymous in view of the very real personal and professional risks involved in providing information on the sensitive topics we discussed. Due to these constraints, we use pseudonyms and anonymous quotes for information collected in confidential interviews. Although several lacunae remain and further research is needed, this approach can help illuminate understudied governance practices so as to better grasp the impact of welfare provision during the Syrian conflict.

## Performing the State: The Importance of Welfare

The importance of welfare services and food distribution during war is well borne out by the historical record (Just and Trentmann 2006; Collingham 2011). Nevertheless, they remain vastly under-studied in analyses of contemporary conflict, largely because of the assumptions still prevalent in the study of civil war. Previously, Hobbesian conceptions of order dominated the literature.^2^ As a result, most scholarship deployed theoretical frameworks that embraced a binary between state-sanctioned order and anarchy. Over the past five years, an impressive amount of the civil war literature has dismantled this paradigm (Wood 2008; Mampilly 2011; Arjona 2014), demonstrating that although internal warfare may fragment political authority, it does not eradicate it altogether (Staniland 2012; Krasner and Risse 2014; Naseemullah 2014). When the monopoly on the legitimate use of force is challenged or ceases to exist, new configurations of political order frequently emerge, as do the public services and everyday institutional practices these can engender (Mampilly 2011, 50; Staniland 2012). Policy-related accounts of the Syrian civil war and our interviews with civilians bolster this claim with regard to this conflict. Yet the analytical tools needed to dissect such empirical circumstances remain underdeveloped (Staniland 2012).

The few accounts of wartime contexts that have embraced concepts such as governance to capture the broader relationships between rulers and ruled frequently retain an implicit attachment to outmoded understandings of the state. The frameworks they employ contain a rigid distinction between the state and society, which are depicted as two concrete entities with discernible boundaries—a characterization that extends to more recent studies of rebel governments (Weinstein 2006; Metelits 2009; Peksen and Teydas 2012; Arjona 2014). The empirical challenge of apprehending the state has fostered a form of theoretical abstraction that serves to reproduce a conception of the state as a concrete institutionalization of power with faculties that can be operationalized.^3^ State capacity or power is then measured according to the ability of official institutions to extract revenue (Bhavnani and Snyder 2005; Besley and Persson 2008, 2010; Thies 2010), provide certain services (Thyne 2006; Peksen and Taydas 2012), or deploy coercion (DeRouen and Sobek 2004; Boix 2008). Although this approach allows for an analysis of the provision of public goods, distributive efforts are catalogued simply as observable or quantifiable components of state capacity (Azam 2001; Burgoon 2006; Fjelde and De Soysa^2009^; Peksen and Taydas 2012). Similar assumptions color more recent works analyzing rebel governance (Weinstein 2006; Metelits 2009; Barter 2015).

By ascribing to the state an institutional identity and an assumed coherence, most contemporary accounts of civil war obscure the processes through which political authority is performed and experienced. In doing so, they lose sight of the micro practices, symbolic acts, and everyday techniques that give a governing authority both its institutional appearance and its power (Mitchell 1991, 77–96; Mampilly 2015, 74–95). Analyses of civilian life and politics in conflict zones become limited when disregard for the diverse nature of ruler-ruled relations combines with this overly top-down and institutionalist reading of the state. To avoid these shortcomings, we believe civil war scholars must follow their peers in other fields and reconceptualize the state and rebel governing institutions not as a discernible organizational entity or a natural expression of political authority but as contingent political constructs—a “structural effect” of power (Mitchell 1991). Although variants of this approach have been employed in a host of colonial, postcolonial, and post-conflict situations (Mitchell 2002; Wedeen 2009; Jeffrey 2012; Neep 2012), they have been far less influential in the study of civil war.

To expand the study of governance during wartime beyond its merely quantifiable and functionalist aspects, we posit that states do not simply exist, nor are they the stable backdrop to political life. Rather, their legitimacy and ability to rule “rely on a capacity to perform their power” (Jeffrey 2012). Thus, the state is represented, reproduced, and summoned into existence through everyday practices that make it “appear to exist” (Mitchell 1991, 94). Drawing on the work of Alex Jeffrey (2012), we refer to these practices as “performing the state.” Frequently, these acts are not grandiose, lavish, or spectacular, but prosaic (Painter 2006). Examples include the policing of borders, the collection of taxes, and the sanctioning of education textbooks. As Painter argues, these “mundane *practices”* are a crucial element “through which something we label ‘the state’ becomes present in everyday life” (Painter 2006, 753). This article focuses on the production of state effects through the provision of welfare services, with particular emphasis on the distribution of food. We do so because welfare—defined as the direct distribution or indirect facilitation of services, programs, and infrastructure intended to promote the well-being and security of recipients—helps project an image of “infrastructural power” (Mann 1984), which reinforces the omnipresence of governing institutions (Cammett and MacLean 2011). Of course, an explicit material exchange is often involved, but the immaterial dimensions of welfare are equally important. Through repetition, welfare services help political actors foreground their authority within the everyday patterns of social life, making institutions, as well as the authority and sovereignty they seek to consolidate, both tangible and thinkable.^4^ The provision of subsidized bread, for example, helps build community; it signals membership in a polity, while offering security and psychological comfort to local populations, especially among the poorest beneficiaries. Assuring basic needs can also foster goodwill, establish a reputation for reliability, and promote certain visions of group affiliation and political loyalty, while signaling a desire or capacity to govern successfully on a local or national scale.

Welfare is certainly not the only means through which military actors perform the state during civil war. However, these provisions play an important role, especially during wartime, when material resources and security become scarce and the contingent nature of political order becomes increasingly overt. In Syria, bread provision is one of the many ways in which competing military forces bring political authority into being—an everyday occasion and iterative performance that makes their authority and claims to legitimacy palpable to ordinary citizens. Such enactments do not occur in a vacuum, but emerge in the wake of preexisting mechanisms of coercion, moral, and political economies and institutional legacies; they are a product of iterative social practices that “both presuppose and conjure anew distinct visions of community” (Wedeen 2009, 215). Put differently, wartime attempts to perform the state, and their reception, are shaped not just by available material resources but also by prewar legacies as well as the political rationalities and governmental assemblages with which they are enmeshed.

## Tracing Bread’s Importance

Popular protests linked to food shortages have a long history in the Middle East (Gough and Zurayk 2014). World War II proved a crucial turning point for food-related provisions and social policy in the region. Allied forces could ill afford an expansion of the popular unrest that first appeared in Cairo, Beirut, and Damascus after the onset of conflict in Europe (Wilmington 1971, 25). To manage the wartime shipping regime and the distribution of key subsistence goods, British authorities established the Middle East Supply Centre (MESC) in April 1941. This new body organized agricultural production and regulated food distribution, undertaking censuses and surveys to guarantee equitable food provision throughout the region. Regarding bread specifically, an agency known as the Wheat and Cereals Office, working under the auspices of the Spears Mission,^5^ created an integrated system of grain collection, transport, processing, and distribution (Heydemann and Vitalis 2000). Through such efforts, the Allies and their local partners safeguarded food security, which minimized local unrest and fostered social stability (Wilmington 1971; Collingham 2011, 127-31). Ultimately, these and other welfare provisions proved crucial to the Allies’ military success in the Levant (Collingham 2011, 123-31).

The MESC’s incursion marked an important departure from the laissez-faire approach that had shaped social policy in Syria before WWII. For example, an array of previously private bakeries and flour mills were nationalized so as to produce and distribute bread more effectively in large urban centers. Syrian government officials, first exposed to interventionist economic management techniques under the MESC, subsequently integrated a number of analogous measures into the postindependence institutional apparatus (Heydemann 1999, 70). These changes were not simply the product of elite-level dictates—Syrian citizens became co-producers of welfare systems, demanding from political authorities similar forms of support to those that had been provided during WWII.^6^ Post-independence shifts in food policy succeeded because the emergence of interventionist ideas of economic management coincided with consumer protests and appeals (Neep 2015). Following the Ba’ath Party’s ascent to power in 1963, the government expanded its involvement in food markets as part of a “successful consolidation of a radical populist authoritarian system of rule” (Heydemann 1999, 161).

Similar to several of its regional counterparts, the Assad-led Ba’thist regime (1970- ) provided various forms of social welfare to the citizenry. These included free healthcare, education, subsidized food, and utilities. This was part of a tacit social pact famously described by Tunisian scholar Larbi Sadiki as *“dimuqratiyyat alkhubz* (democracy of bread),” in which public goods were provided in exchange for political compliance (Sadiki 1997). Subsidized bread stood at the forefront of these tacit agreements. Since the early 1970s, the Syrian government has supported wheat production by subsidizing most agricultural inputs and paying farmers above market value for their crops^7^. The government finances bread consumption by subsidizing retail prices, which are set far below production costs. Following Bashar al- Assad’s accession to the presidency in 2000, there was a decisive turn away from the previously interventionist model of development. The young president and his loyal coterie undertook measures that moved Syria toward a distorted form of market economy in what amounted to a misshapen process of neoliberalization (Haddad 2011; Hinnebusch 2012). While the proportion of government expenditures allotted to education, public employment, and pensions were reduced considerably, oil and food subsidies remained stable, accounting for nearly 15 percent of government spending prior to the outbreak of protests in 2011 (Breisinger et al. 2012). Not incidentally, official institutions controlled all distribution channels of the country’s “most important staple food” (Chemingui et al. 2010, 159) before the current conflict, acting as the exclusive purchaser of local wheat and sole flour supplier to bakeries producing subsidized bread. Throughout its long tenure, the Assad regime has treated the bread subsidy as an indisputable governmental responsibility toward its citizenry, with various notable officials referring to the policy as a “red line” (SAMA Syria 2014). Undoubtedly, much of this is due to bread’s nutritional importance: before the war, wheat provided approximately 40 percent of Syrian households’ caloric consumption, mostly in the form of bread (FAO 2013). Yet, equally important is bread’s symbolic role as one of the government’s few remaining commitments to what was once an expansive welfare state, a crucial means through which the Assad regime performs the state.

Since the onset of violence in late 2011, the Syrian government has done its best to maintain the bread subsidy in areas it controls by ensuring that bakeries are open, well stocked with flour, and consistently distributing the foodstuff. Interestingly, various opposition groups have sought to gain civilian support by mimicking elements of the government’s welfare programs. Like the Assad regime, they interact and negotiate with local populations in exchange for their loyalty or compliance.^8^ For example, during the first two years of the conflict, activist-run and democratically elected civilian authorities such as the Transitional Revolutionary Council of Aleppo used donations from Gulf countries to pay for flour and bread distribution before funding other projects, such as salaried police (Amos 2012).

More recently, after capturing Idlib city in 2015, a coalition of Islamist and FSA- affiliated rebels immediately sought to restore the provision of baking supplies to civilians after shortages caused by fighting (Al-Aan TV 2015; Abdulrahim 2015). Upon taking control of Palmyra from government forces in 2015, the Islamic State immediately reopened the city’s only remaining bakery and began distributing bread for free, a tactic it has frequently employed when it conquers new territories (Barnard and Saad 2015). Pre-conflict practices and civilian expectations have intimately shaped the behavior of both the government and rebel groups. They are crucial factors explaining bread provision’s continuing importance (Mampilly 2015). At the same time, not every military faction deems welfare infrastructures to be worthy of time and effort; service provision is a strategic decision based on each group’s respective resources and goals. Throughout the war, the Assad regime, the Free Syrian Army (FSA), and the Islamic State (IS) have tended to rely less on generating durable institutions or consistent welfare services to perform the state than on sporadic demonstrations of their coercive power, especially in contested areas. Bread provision remains a notable exception.

## Bread Is a Red Line: The Assad Regime’s Wartime Welfare Strategies

Faced with the private sector’s retreat since the beginning of the conflict in 2011, the Syrian government has expanded its role in the lives of civilians under its control (Khaddour 2015). Central to such interventions is the provision of sustenance goods (water, electricity, fuel), including subsidized bread. Distribution of the latter has been complicated during the ongoing crisis. Syria was self-sufficient in wheat before the war, but over the past four years local production has collapsed. In 2014, the country’s cereal harvest was 50 percent below prewar levels and wheat production was at its lowest in 25 years (FAO 2015; Reuters 2015a). Out of the 140 wheat collection centers in operation before the conflict, only forty remain. Four of the country’s five yeast factories have shut down entirely. Damaged wheat silos, destroyed flour mills, poor harvests, transportation impediments, and military operations have forced the General Establishment for Cereal Processing and Trade (HOBOOB)—the agency of the Syrian Ministry of Supply and Internal Trade responsible for wheat procurement, flour milling, and timely bread provi- sion—to alter its distributive strategies (Adan 2014). Additionally, widespread looting coupled with combat-related destruction to warehouses has drastically reduced storage capacities (Syria Relief and Development 2014). In 2015, the government claimed to have only one million tons of wheat left in storage silos, whereas before the war it kept three million (Reuters 2015b). The economic crisis caused by the ongoing war further complicates the accessibility and affordability of essential foodstuffs, as general food prices rose by an estimated 131 percent from 2011 to 2015 (WFP 2015b). The local currency’s depreciation combined with fuel shortages has made bread production increasingly expensive (FAO 2015; Sotloff 2013). Civilians in opposition-controlled territory have been the most affected. By March 2015, bread prices in some areas rose by 300 percent on average and up to 1000 percent in certain highly contested locales (WFP 2015a).

To maintain the bread subsidy amid rising costs, the Syrian government has turned to its foreign backers for help. Since the implementation of economic sanctions in mid-2011, the government has been forced to use smaller ships and pay higher prices for the import of comparatively modest quantities of wheat. Frequently, Lebanese traders act as middlemen in these transactions in order to help conceal the ships’ final destination, as larger banks, shipping companies, and grain traders are wary of the Assad regime’s credit (Reuters 2012; Al-Khalidi 2013). On other occasions, the government has used foreign assistance to purchase wheat from local farmers in opposition-controlled areas by paying higher premiums than private traders or rebel groups (Al-Khalidi 2015). It has also repeatedly used a $3.6 billion credit line furnished by Iran in 2013 to issue commercial tenders, not for arms or mercenaries, but for wheat (Lucas 2013). Prominent government officials promised to use a second $1 billion Iranian credit line issued in 2015 to “secure the flow of essential goods and materials” (Al-Khalidi and Westall 2015). Despite two separate increases in subsidized bread prices in 2014 and 2015 and a host of logistical challenges, bread remained readily available in large swathes of territory under regime jurisdiction from 2012 to 2015. Assad’s wartime cabinet was cognizant that a failure in food supplies would impact not only the army and important war industries but, crucially, civilian morale. “We will always provide the needs to produce bread, which is a red line for the syrian government,” said Syrian Prime Minister Wael Halqi in 2014 (*Syrian Observer* Ensuring the availability of food at affordable prices, he added, was one of the government’s highest priorities (*Syrian Observer 2014*). Less than one year later, Syria’s Minister of Domestic Trade and Consumer Protection, Hassan Safiyeh, stated that President Assad himself had told him to secure the basic necessities of the citizenry “regardless of the circumstances” (Bassam and Perry 2015). To meet this objective despite the aforementioned challenges, the regime has increased its spending threefold on the bread subsidy since 2012 (SANA 2015c; Bassam and Perry 2015). By shouldering the costs of this increasingly expensive welfare program and ensuring its timely execution, the Assad regime performs the state, presenting itself as the protector of the citizenry and the guarantor of its well-being.

The Assad regime has consistently used welfare provision to win popular support in areas of contested or joint rule. During a 2015 flare-up between government and Kurdish Peoples Protection Units [YPG] forces in Al-Hasakah city—the capital of the eponymous province over which the two parties jointly rule—the government made a concerted effort to alleviate a bread crisis caused by the hostilities (Yekiti Media 2015). Amid the deteriorating security situation in the city, the Assad-backed municipal administration announced that it would keep bakeries open on weekends to satisfy customers’ needs (SANA 2015b). Emphasizing the importance of “providing public services and basic needs to citizens in order to overcome the difficult circumstances,” the regime-affiliated governor assured civilians on Syria’s official state news agency that bakeries were running smoothly following the government’s delivery of 150 tons of flour to loyal parts of the city (SANA 2015b). In response, the YPG set up its own bread distribution apparatus (Abu Zeid and Eng 2015). “The product of our [bakeries] is more desirable than the government bakeries, which are very bad,” said a representative of the PYD, the YPG’s political branch (Ara News 2015). By publicizing its distributive efforts while fighting the regime, the YPG—which otherwise relied heavily on the Syrian government to provide an array of public services in areas it ostensibly controls —sought to demonstrate its ability to provide bread without regime assistance (Lund 2015). The race to provide bread in Al-Hasakah underlines a greater struggle between the Assad regime, which performs the state so as to signal its continuing power to civilians, and an emergent political group that seeks legitimacy by mimicking such state performances.

The provision of bread to regime-controlled areas has gone hand in hand with targeted efforts to deprive rebel groups of the essential foodstuff and, by extension, their ability to perform the state (Eng and Martínez 2014). Since 2012, the regime has bombed nascent opposition-administered attempts to provide vital public services and subsistence goods, thereby presenting itself as the only viable source of such necessities (Khaddour 2015). Bakeries in particular have been systematically targeted. In August 2012, Human Rights Watch (HRW) reported at least 10 government aerial attacks over a three-week period on the bakeries of Aleppo, the center of opposition resistance at the time (Human Rights Watch 2012). Another report released in early 2013 independently verified 80 regime attacks on bakeries between August 2012 and January 2013 (Gutman and Raymond 2013). When the Free Syrian Army and other rebels thrived in the regions of Idlib, Homs, and Deir e-Zor, bakery bombings quickly followed. One former resident of rebel-controlled Aleppo, who had recently fled to Lebanon, highlighted the toll of regime tactics: “The regime has made daily life impossible. How can you send your kids to school, plan for their future or run your business when you do not know if there will be anything to eat the next day?” (Anonymous 6 2015). More recently, the regime has shifted its attention to the Islamic State’s successful bread-making operation (Reuters 2014). In targeting bakeries, the Assad regime limits the ability of opposition parties to execute emblematic state performances. This prevents relations between incipient rebel governing bodies and civilians from being formalized or stabilized. At the same time, the Assad regime’s provision of basic foodstuffs in territories it controls alleviates economic stress, averts popular unrest, and boosts morale among weary civilians, while subtly reminding them of the benefits of state power and administration.

Despite the tremendous measures that the Syrian government has undertaken to continue subsidizing bread, wartime circumstances have slowly altered its calculus. “We live in exceptional circumstances that require exceptional actions and exceptional decisions,” said Hassan Safiyeh after the second official government increase in bread prices during early 2015 (SANA 2015a). Whereas civilians would likely have contested this measure prior to the war, many in regime- controlled territories accepted the change without major protest (Syria Direct 2015a). Indeed, the large majority of our interviewees acknowledged that in comparison to opposition-controlled areas of the country, the foodstuff remains both widely available and comparatively cheap. Many respondents spoke highly of the government’s continuing efforts to ensure the provision of sustenance goods. For example, one resident of Damascus’ Bab Sharqi neighborhood stated: “The regime clearly does its best to provide bread. Given the precarious economic situation and the war, we feel the price increase is justifiable. Bread is still cheaper than Lebanon or areas under opposition control” (Anonymous 4 2015). However, there is also evidence that citizens are not unanimously tolerant of all changes to the sacrosanct welfare program. One clear example was popular protests in Al-Hasakah and Damascus in March 2015, during which citizens denounced declining bread quality (Orient News 2015a). The rapid palliative measures that the regime took to address protestor demands point to the ways in which local residents continue to play an active role in ensuring that the government does not renege on its perceived responsibilities, even as wartime circumstances alter those obligations (Orient News 2015a, 2015b; Reuters 2015b). Welfare practices during civil war are hardly static; they respond and react to available resources, civilian demands, and political-military calculations. Subsidized bread is part of a wider supply chain and production process that is not easily sustained, reliant as it is on a rather precise combination of water, flour, and yeast.^9^ Performing the state is a process continually in motion.

## Underperforming: FSA Governance in Aleppo

Bread provision was crucial to FSA-affiliated rebels’ failure to maintain support in Syrian communities. Although military setbacks in 2013 and 2014 turned many against this loose amalgamation of fighting battalions, its dismal track record in local administration also played a significant role in the FSA’s decline.

Throughout 2013, widespread shortages of basic necessities in areas under FSA control led to massive price increases and thriving black markets, much to the frustration of local residents (Syria Frontline 2012). In early January, one couple in Aleppo stated that “We used to live in peace and security until this malicious revolution reached us and the FSA started taking bread by force” (Bayoumy 2013. An activist in Idlib offered similar criticism of the FSA, claiming that “(they) did not provide [security, food, water, electricity and medicine] to civilians. They only provided support to their soldiers, they weren’t interested in anyone else” (Syria Direct 2014c). FSA-affiliated groups were widely accused of capturing wheat silos and selling grains abroad and on black markets rather than distributing them among the citizenry (Sotloff 2013; Syria Direct 2014c). In various interviews, residents of Idlib complained of increases in the prices of fuel, flour, and other foodstuffs, which they blamed on FSA corruption. “They stole our revolution,” said one resident of Aleppo who had fled to Beirut. “By taking our bread and making everyday life impossible they lost our support” (Anonymous 7 2015).

The link between the FSA’s failure to perform the state and its military collapse is most evident in the city of Aleppo. Earlier in the conflict, FSA fighters worked with local coordination committees (LCCs)—democratically elected Syrian National Coalition-affiliated governance councils—to supply wheat, flour, and other inputs to bakeries, which then produced and sold bread at subsidized prices. Through their focus on welfare services and humanitarian assistance, LCCs attempted to fill a gap that military forces such as the FSA often could not initially address.^10^ In late 2012, a collection of activists and professionals called the Transition Revolutionary Council in Aleppo city received $1 million from Qatar, which immediately went toward the purchase of wheat to relieve a bread crisis (Amos 2012). This project took precedence over a host of other pressing concerns and was described by one interviewee as a “crucial part of the FSA’s initial success amongst local residents” (Anonymous 2 2013). Yet over time, the FSA’s failures to distribute or facilitate the provision of affordable bread to residents of Aleppo caused many to lose faith in the armed group. FSA fighters were known for regularly cutting lines and taking the bulk of available food for themselves (Sotloff 2013). Social media outlets affiliated with Islamist groups published statements accusing the FSA of rampant corruption. Simultaneously, they publicized their own efforts to efficiently provide basic materials to local communities (Hassan and Weiss 2015, 221-33). Interviewees regularly cited the administrative failures and regular abuse of authority by the FSA as crucial to changes in the balance of power in the city. The rebel group was described “as being unable to establish livable spaces,” despite its attempts to incorporate the LCCs into its governance efforts (Meininghaus 2016). This story was repeated throughout Syria, as the FSA’s administrative failures demoralized and estranged a population initially inclined to support its cause (for similar findings, see Hassan and Weiss 2015, 222-26).

When the FSA’s shortcomings became clear in late 2012 and 2013, Islamist factions began to gain a foothold throughout the country, especially in northern Syria. “The so-called terrorists are the ones who have been giving us bread and distributing it fairly,” said Tamam Hazem, a spokesperson for one of Aleppo’s news centers (MacFarquhar 2012). “[Islamist groups] are offering bread to people to obtain their sympathy and respect,” said Jalal al-Khanji, Aleppo city’s local council president in December 2012 (MacFarquhar 2012). The Islamists proved more effective in both governance and battle than the disorganized FSA militias, and were often welcomed by local populations (Cockburn 2015, 85). By mid-2013, various groups had strengthened their control through the efficient distribution of public goods: “The new forces understand that we cannot live without bread, they use this to their advantage,” a resident of Aleppo stated at the time (Anonymous 1 2013). Groups such as Jabhat al-Nusra and the Islamic State (IS) excelled where the FSA had failed—the provision of food and physical security—earning reputations as disciplined and reliable governing authorities (Khatib 2015). These efforts were crucial to winning popular support in Aleppo throughout 2013.

## Performing the Islamic State

While observers attribute much of the Islamic State’s meteoric rise to its string of military victories and clever social media tactics, the organization’s efforts to provide bread, security, and other basic services were crucial to its initial expansion in Syria. One of the organization’s first steps upon taking over a town was to control key industries and services related to electricity, water, fuel, and bread in order to assert total control over the core needs of the local population (Lister 2015, 47). In addition, after IS expelled rival factions from a locality, such as the Liwa Ahrar al-Jazira brigade from the Eastern border town of Yaroubiya in Al- Hasakah in mid-2013 or the Northern Storm battalion from Azaz in Aleppo in late 2013, its administrators subsequently lowered the price of bread to undermine support for rival groups (Al-Tamimi 2014, 10). In the strategically located city of Manbij in Aleppo province, IS’s takeover was quickly followed by the establishment of a “full-fledged system of governance, impressing city denizens and displaced persons alike” (Hassan and Weiss 2015, 223). The group’s effectiveness in resolving disputes and providing services such as sanitation and food delivery made it appear as the only alternative to societal collapse. This led many civilians, at least initially, to accept the imposition of IS’s despotic measures (Lister 2015, 46-49).

As part of its state-building project proclaimed in its well-known slogan *baqiya wa tatamadad* (lasting and expanding), IS published a pamphlet in 2014 outlining the services it offers to the province of Aleppo, where it has intermittently governed pockets of territory since September 2013 (Wilayat Halab 2014). The publicity pamphlet describes various facets of its administration and includes all of the services one might expect from a local government: distribution of water, collection of taxes, and electricity installation. The document also outlines plans to plant and harvest wheat in coming years and underlines the organization’s efforts to “manage bakeries and mills to ensure access to bread for all” (Wilayat Halab. This was not mere rhetoric. In Deir e-Zor, IS reduced the price of bread from 200 to 45 Syrian pounds and made it mandatory for bakeries to offer charitable contributions to the poor after taking over most of the province in 2014 (Hassan 2014; Karouny 2014; Lister 2015, 48-49). IS directly subsidized the cost of flour, accelerated the opening of bakeries, and distributed bread itself when necessary, demonstrating a keen focus on “the essential ingredient to gaining popular support” (Syria Direct 2014b). In Raqqa, IS did much the same. “Daily life in the city is good,” said Abu al-Bara’a al-Furati, a member of the Raqqa Media Center in 2014. “I’ll start with the most important thing for us, which is bread—there is a surplus of bread available in the city, more than bakeries need” (Syria Direct 2014a). Initially, IS’s model of governance proved successful in many war-ravaged and food-insecure Syrian towns and villages (Lister 2015, 26).

Since August 2014, a US-led aerial campaign has worked to destroy and undermine the organization’s revenue streams in Iraq and Syria, which has had a notable impact on IS’s welfare services. In early 2015, IS raised the price of bread to more than three times its cost prior to its takeover of Raqqa, from 4 SP for a single loaf to an “unprecedented” cost of 12.5 SP (All4Syria 2015). IS’s failure to provide for those living under its authority has weakened its position in many areas it controls, leading the group to increasingly rely on force to maintain its hold. Public opinion has grown increasingly critical in response. “When it first entered [Deir e-Zor], IS undertook some simple works to attract attention and popular support,” said Abu Mujahed a-Sharqiya, a media activist in the IS- controlled city of al-Mayadin (Syria Direct 2015b). “But after it learned about the oil profits in Deir e-zor, it became solely interested in running the oil wells and collecting money.” Since late 2014, local residents have likewise accused IS of transferring wheat from Deir e-zor and Raqqa—two areas known for their agricultural fecundity prior to the war—to Iraq, where they sell it to traders to fund their military operations instead of supplying local bakeries (Arabi21 2015).

While bread provision helped IS gain a foothold in opposition-held areas previously dominated by other rebel groups, its subsequent inability or unwillingness to continue performing the state loosened its hold over several Syrian communities. In Raqqa and Deir e-Zor, IS’s failure to provide basic goods has fostered resistance among a population increasingly unwilling to tolerate its erratic rule (Raqqa Is Being Slaughtered Silently 2015). Popular discontent with the group has helped foster the formation of several militia brigades that have conducted clandestine attacks and assassination attempts against IS members, suggesting increasing boldness on the part of civilians (Anonymous 3 2015). In the face of military threats and financial pressures, IS prioritized its military capacity and resources at the expense of its previously robust distributive apparatus (Ensign and Shaver 2015). Although the former helped IS maintain control in places like Raqqa and Deir e-Zor, there was increasing dissent among inhabitants living under its control throughout 2015. Ironically, IS repeated some of the very same FSA mistakes that facilitated its emergence.

## Conclusion

In Syria, food provision is a politicized landscape. The FSA’s inability to adequately supply bread and other basic necessities contributed to its loss of popular support and eventual fade into irrelevance in 2013 and 2014, especially in Northern Syria (Al-Tamimi 2014, 8; Cockburn 2015, 114). Where FSA-affiliated battalions floundered, hardline Islamist groups such as IS and Jabhat al-Nusra flourished. Not only did they offer various welfare services effectively, they were also far more successful in publicizing their efforts, which bolstered their image as civilian-conscious rulers. The onset of American-led airstrikes in late 2014 and subsequent financial and military challenges, however, has led IS to retract some of its services. IS’s shift away from effective welfare provision, and the ensuing loss of local support, suggest both the difficulty of consolidating the infrastructure necessary to “perform the state” during conflict, as well as the importance of consistent provision in doing so. other Syria-based Islamist organizations, such as Ahrar al-Sham and Jaish al-Islam, have tried to position themselves between these two poles. Many of these groups have worked with, and sometimes pressured, LCCs to bring in international humanitarian aid and provide welfare services in a bid to foster support among local residents (Martínez and Eng 2016). Those most adept at these undertakings endure as important players on the battlefield. As poverty levels and food insecurity increase, civilians have shifted their allegiances to actors best capable of ensuring their basic needs (Chingono 1996; Lubkemann 2005). It is thus no surprise, then, that the Assad regime has focused on disrupting rebel attempts to perform the state.

our analysis has attempted to demonstrate the importance of bread provision to the Syrian conflict. Although there is no one factor that can singularly explain the emergence, development, or impact of welfare services during civil war, we have tried to highlight key reasons for their importance. Undoubtedly, further research is needed. Yet, by conceptualizing the state as a construct, an effect of power, partially produced through everyday performances such as the distribution of public goods, we believe scholars can better understand the role, impact, and salience of welfare provision, especially during civil war. Such an approach can also help illuminate the otherwise opaque relations between welfare provision, military success, and civilian agency during civil war. While the provision of subsistence goods may initially appear to be an epiphenomenal component of the Syrian conflict, it has proven critical to its development. That being said, food provision is only one of many intersecting variables that will influence the war’s outcome. What we can say with some certainty, based on our admittedly brief look into the conflict, is that welfare services are prominent in the calculations of all the forces hoping to rule Syria, and that subsidized bread stands at the forefront of these concerns.

Authors’ note: The authors are grateful to Zachariah Mampilly, Alex Jeffrey, Mark Duffield, Lewis Turner, José Darío Martínez, Ali Nehme Hamdan, Lizzie Presser, and Abdulqader Dali for their comments, critiques, and suggestions. They would also like to thank the many Syrians who offered their time and support, but who have asked to remain unidentified.

## Footnotes

1 These include but are not limited to the pre-conflict scope of government institutions and their legacy, the internal and external political agendas of military forces, competition for territorial control, relationships with transnational and local actors, and coercive capacities (Mampilly 2011, 16, 234).

2 For an excellent critique of this inclination, see Mampilly (2011).

3 For a survey of this literature, which concludes by defending a multivariate approach to modeling state capacity, see Hendrix (2010).

4 We conceive of sovereignty not as a container concept but as a specific “political order produced by an assemblage of administrative strategies,” performed and planned to generate allegiance, fear, compliance, and legitimacy from the household to the highest echelons of institutional power (Hansen and Stepputat 2005, 297; Ong 2006, 80).

5 In Syria and Lebanon, the MESC operated under the auspices of the Spears Mission, an organization established by Britain to administer the country’s wartime relationship with the French mandates.

6 The terms of this new social pact were institutionalized in Syria’s 1950 constitution, which included prominent references to social welfare, land reform, and peasants’ rights (Heydemann 1999, 30-84; Heydemann and Vitalis 2000, 106).

7 The UN embargo on Iraq following the invasion of Kuwait galvanized Syria’s wheat self-sufficiency campaign in the early 1990s. Hafez al-Assad feared the repercussions of a potential UN embargo on the country’s food supply.

8 Of course, not all civilians decide to exert agency by pressuring local governing forces; many Syrians have chosen instead to flee abroad or to safer areas in the country.

9 We thank Alex Jeffrey for bringing this point to our attention.

10 For more on the relationship between civilian bodies and military groups in opposition-held areas, see Baczko, Dorronsoro, and Quesnay (2013); Yassin-Kassab and Al-Shami (2016, 77-146).
